# The variant call format provides efficient and robust storage of GWAS summary statistics

**DOI:** 10.1186/s13059-020-02248-0

**Published:** 2021-01-13

**Authors:** Matthew S. Lyon, Shea J. Andrews, Ben Elsworth, Tom R. Gaunt, Gibran Hemani, Edoardo Marcora

**Affiliations:** 1 National Institute for Health Research (NIHR) Bristol Biomedical Research Centre (BRC), Bristol Medical School (Population Health Sciences), University of Bristol, Oakfield House, Bristol, BS8 2BN UK; 2 Medical Research Council (MRC) Integrative Epidemiology Unit (IEU), Bristol Medical School (Population Health Sciences), University of Bristol, Oakfield House, Bristol, BS8 2BN UK; 3 Ronald M. Loeb Center for Alzheimer’s Disease, Department of Neuroscience, Icahn School of Medicine at Mount Sinai, New York, NY 10029-5674 USA

**Keywords:** GWAS, VCF, Summary statistics, Storage format

## Abstract

GWAS summary statistics are fundamental for a variety of research applications yet no common storage format has been widely adopted. Existing tabular formats ambiguously or incompletely store information about genetic variants and associations, lack essential metadata and are typically not indexed yielding poor query performance and increasing the possibility of errors in data interpretation and post-GWAS analyses. To address these issues, we adapted the variant call format to store GWAS summary statistics (GWAS-VCF) and developed open-source tools to use this format in downstream analyses. We provide open access to over 10,000 complete GWAS summary datasets converted to this format (https://gwas.mrcieu.ac.uk).

**Supplementary Information:**

The online version contains supplementary material available at 10.1186/s13059-020-02248-0.

## Background

The GWAS is a powerful tool for identifying genetic loci associated with any trait, including diseases and clinical biomarkers, as well as non-clinical and molecular phenotypes such as height and gene expression [1] (eQTLs). Sharing of GWAS results as summary statistics (i.e. variant, effect size, standard error, *P* value) has enabled a range of important secondary research applications including causal gene and functional variant prioritisation [2], causal cell/tissue type nomination [3], pathway analysis [1], causal inference (Mendelian randomisation (MR)) [4], risk prediction [1], genetic correlation [5] and heritability estimation [6]. However, the utility of GWAS summary statistics is hampered by the absence of a universally adopted storage format and associated tools.

Historic lack of a common standard has resulted in GWAS analysis tools outputting summary statistics in different tabular formats (e.g. plink [7], GCTA [8], BOLT-LMM [9], GEMMA [10], Matrix eQTL [11] and meta-analysis tools, e.g. METAL [12]). As a consequence, various processing issues are typically encountered during secondary analysis. First, there is often inconsistency and ambiguity of which allele relates to the effect size estimate (the “effect” allele). Confusion over the effect allele can have disastrous consequences on the interpretation of GWAS findings and the validity of post-GWAS analyses. For example, MR studies may provide causal estimates with incorrect effect directionality [13]. Likewise, prediction models based on polygenic risk scores might predict disease wrongly or suffer reduced power if some of the effect directionalities are incorrect. Second, the schema (i.e. which columns/fields are included and how they are named) of these tabular formats varies greatly. Absent fields can limit analyses, and although approaches exist to estimate the values of some of these missing columns (e.g. standard error from *P* value), imprecision is introduced reducing subsequent test power. Varying field names are easily addressed in principle, but the process can be cumbersome and error-prone. Third, data are frequently distributed with no or insufficient metadata describing the study, traits and variants (e.g. trait measurement units, variant ID/annotation sources) which can lead to errors, impede the integration of results from different studies and hamper reproducibility. Fourth, querying unindexed text files is slow and memory inefficient, making some potential applications computationally infeasible (e.g. systematic hypothesis-free analyses).

Some proposals for a standard tabular format have been made. The NHGRI-EBI GWAS Catalog (www.ebi.ac.uk/gwas) developed a tab-separated values (TSV) text format with a minimal set of required (and optional) columns along with standardised headings [14]. The SMR tool [15] introduced a binary format for rapid querying of quantitative trait loci. These approaches are adequate for storing variant level summary statistics but do not enforce allele consistency or support embedding of essential metadata. Learning from these examples and our experiences performing high-throughput analyses across two research centres, we developed a set of requirements for a suitable universal format. We determined that adapting the variant call format (VCF) [16] was a convenient and constructive solution to address these issues. We provide evidence demonstrating how the VCF meets our requirements, showcase the capabilities of this medium and introduce tools and resources for working with this format.

## Results

### Requirements

Our requirements for a universal GWAS summary statistics format specification were developed through the experience of collecting and harmonising GWAS summary data across two research centres at scale (Table 1). These features place emphasis on consistency and robustness, capacity for metadata to provide a full audit trail, efficient querying and file storage, ensuring data integrity, interoperability with existing open-source tools and across multiple datasets to support data sharing and integration.

**Table 1:** Requirements for a summary statistics storage format and solutions offered by the VCF

Requirement	Solution using the variant call format
Human readable and easy to parse	Read with any text viewer. Mature open-source parsing libraries are available (HTSLIB [17] and HTSJDK [17]) and implemented in most modern programming languages, for example, VariantAnnotation [18] R-package is available from Bioconductor [19–21] and Python package pysam [17, 22]. Bcftools [23], GATK [24], bedtools [25] and others provides user-friendly functionality from the command line.
Unambiguous interpretation of the data	Data field descriptions, value types and number of values are required and defined in the file header. File validity is enforced during each read/write.
Unambiguous representation of bi-allelic, multiallelic and insertion-deletion variants	Every variant substitution is represented by reference and alternative allele haplotypes defining the exact base change on the forward strand. The reference allele is required to match genome sequences defined in the file header. The alternative allele is always the effect allele allowing consistency between studies for ease of comparison.
Genomic information can be validated	The file header contains information about reference genome assembly and contigs. Reference alleles must match the sequence in the referenced genome build (in FASTA format). GATK [24] ValidateVariants can be used to verify file format validity and compare reference allele information against the corresponding genome reference sequence.
Flexibility on which GWAS fields are recorded and enforcement of essential fields	All fields are defined in the file header and can be set optional or required as desired. The specification contains essential fields and their reserved names.
Capacity to store metadata about the study and traits	The file header contains information about the source and date of summary statistics, study IDs (e.g. PMID/DOI of publication describing the study, or accession number and repository of individual-level data), description of the traits studied (e.g. type, association test used, and measurement unit) as well as the source and version of trait IDs (e.g. IEU OpenGWAS database [26], Experimental Factor Ontology [27], Human Phenotyping Ontology [28], Medical Subject Headings [29], IDs for clinical and other traits, Ensembl Gene IDs for eQTL datasets or any other ontology to describe the data).
Allows multiple traits to be stored together	The SAMPLE column was chosen to store variant-trait association data to allow for storage of multiple traits in a single VCF file or as individual files if desired.
Rapid querying by variant identifier, genomic position interval or GWAS summary statistics value (range or exact value)	The file is sorted karyotypically and indexed by chromosome position using tabix [30] to enable fast queries by genomic position. Secondary indexing on dbSNP [31] identifier is also provided using rsidx [32]. Refer to performance comparisons of indexed VCF files and standard UNIX tools.
File compression	VCF files may be compressed with block GZIP [23] or converted to a binary call file which is a binary VCF companion format [23].
Readable by existing open-source tools	A large number of tools support VCF files including GATK [24], Picard [33], bcftools [23], bedtools [25], vcftools [16] and plink [7]. Bcftools [23] can also provide a tabular extract for use with non-compatible tools.
Amenable to cloud-based streaming and database storage	Genomic intervals may be extracted over a network using a range request which extracts file segments without transferring the whole file. This enables rapid streaming of queries over the Internet. For high-throughput and distributed storage and querying, VCF files can be easily imported into GenomicsDB [34].

*GWAS* genome-wide association study, *dbSNP* database of single-nucleotide polymorphisms, *HTSLIB* high-throughput sequencing data library, *HTSJDK* high-throughput sequencing data Java development kit, *GATK* genome-analysis toolkit, *dbSNP* single nucleotide polymorphism database, *eQTL* expression quantitative trait loci

### File format

The VCF is organised into three components: a flexible file header containing metadata (lines beginning with ‘#’) and a file body containing variant-level (one locus per row with one or more alternative alleles/variants) and sample-level (one sample per column) information. We adapted this format to include GWAS-specific metadata and utilise the sample column to store variant-trait association data (Additional file 1: Fig. S1; Additional file 1: Table S1).

According to the VCF specification, the file header consists of metadata lines containing (1) the specification version number, (2) information about the reference genome assembly and contigs and (3) information (ID, number, type, description, source and version) about the fields used to describe variants and samples (or variant-trait associations in the case of GWAS-VCF) in the file body. We take advantage of the VCF file header to store additional information about the GWAS including (1) source and date of summary statistics, (2) study IDs (e.g. PMID/DOI of the publication describing the study, or accession number and repository of individual-level data) and (3) description of the traits studied (e.g. type, association test used, sample size, ancestry and measurement unit) as well as the source and version of trait IDs (e.g. Experimental Factor Ontology [27], Human Phenotyping Ontology [28], Medical Subject Headings [29] IDs for clinical and other traits, Ensembl gene IDs for eQTL datasets or any other ontology or identifier).

Unlike VCF where a row can contain information about multiple alternative alleles observed at the same site/locus (and thus may store more than one variant), the GWAS-VCF specification requires that each variant is stored in a separate row of the file body. Each row contains eight mandatory fields: chromosome name (CHROM), base-pair position (POS), unique variant identifier (ID), reference/non-effect allele (REF), alternative/effect allele (ALT), quality (QUAL), filter (FILTER) and variant information (INFO). The ID, QUAL and FILTER fields can contain a null value represented by a dot. Importantly, the ID value (unless null) should not be present in more than one row. The FILTER field may be used to flag poor-quality variants for exclusion in downstream analyses. The INFO column is a flexible data store for additional variant-level key-value pairs (fields) and may be used to store for example population frequency (AF), genomic annotations and variant functional effects. We also use the INFO field to store the dbSNP [31] locus identifier (rsid) for the site at which the variant resides. This is because (despite their common usage as variant identifiers) rsids uniquely identify loci (not variants!) and thus cannot be used in the ID field, as we will discuss further at the end of this manuscript. Following the INFO column is a format field (FORMAT) and one or more sample columns which we use to store variant-trait association data, with values for the fields listed in the FORMAT column, for example, effect size (ES), standard error (SE) and -log10 *P* value (LP).

### Query performance

**Fig. 1:**
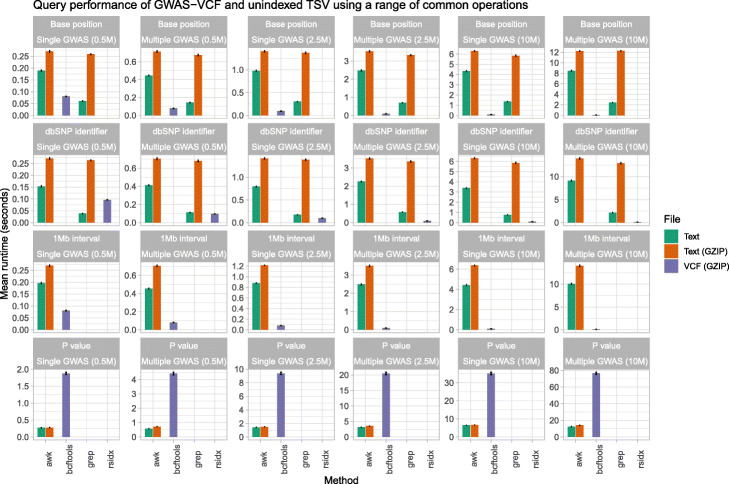
Performance comparison for querying summary statistics in plain text and GWAS-VCF. Mean query time (seconds, lower is quicker; repetitions *n* = 100) to extract either a single variant using the chromosome position or dbSNP [31] identifier or multiple variants using a 1-Mb interval or association *P* value. AWK, grep, bcftools [23] and rsidx [32] were evaluated using uncompressed/GZIP compressed TSV and BGZIP [23] compressed VCF. The summary statistics files contained one (single) or five (multiple) GWAS studies which were prepared by subsampling variants (*n* = 0.5 M, 2.5 M, 10 M) obtain from Neale et al. [35]. Error bars represent the 95% confidence interval

Simulations of query performance demonstrate compressed GWAS-VCF is substantially quicker than unindexed and uncompressed TSV format for querying by genomic position when the GWAS is densely imputed (Fig. 1). The greatest improvements were seen when the GWAS-VCF contained a single trait with 10 million variants where on average GWAS-VCF was 15× faster to extract a single variant using chromosome position (mean query duration in GWAS-VCF 0.09 s [95% CI 0.08, 0.09] vs mean query duration in TSV 1.35 s [95% CI 1.34, 1.37]) and 8x quicker using the rsid (0.1 s [95% CI 0.1, 0.1] vs 0.76 s [95% 0.75, 0.78]). Using a 1-Mb window of variants, GWAS-VCF was 44× quicker (0.1 s [95% CI 0.1, 0.11] vs 4.43 s [95% CI 4.36, 4.5]). Although querying on association *P* value was faster using TSV (mean query duration in TSV 6.48 s [95% CI 6.38, 6.57] vs mean query duration in GWAS-VCF 35.11 s [95% CI 34.35, 35.86]). However, when the number of variants stored in the GWAS-VCF was 0.5 million, uncompressed text was faster for single position and rsid lookups but not interval queries (Fig. 1). Additionally, storing multiple traits in a single GWAS-VCF reduced the *P* value query performance but had little impact on the positional queries (Fig. 1).

### Software

To automate the conversion of existing summary statistics files to the GWAS-VCF format, we developed the open-source Python3 software (Gwas2VCF; Additional file 1: Table S2). The application reads in metadata and variant-trait association data using a user-defined schema requiring the chromosome base-position to start at one. During processing, variants are harmonised using a supplied reference genome file to ensure the non-effect allele matches the reference sequence enabling consistent directionality of allelic effects across studies. Insertion-deletion variants are left-aligned and trimmed for consistent representation using the vgraph library [36]. Finally, the GWAS-VCF is indexed using tabix [30] and rsidx [32] which enable rapid queries by genomic position and rsid, respectively. We have developed a freely available web application providing a user-friendly interface for this implementation (http://vcf.mrcieu.ac.uk/) and encourage other centres to deploy their own instance (Additional file 1: Table S2).

Once stored in a GWAS-VCF file, summary statistics can be read and queried using R or Python programming languages with our open-source libraries (Additional file 1: Table S2) or from the command line using, for example, bcftools [23], GATK [24] or bedtools [25]. These tools also enable variant annotation and filtering (e.g. allele frequency, functional effect, gene and pathway), mapping between reference genome assemblies, file validation and converting to any other tabular format including the NHGRI-EBI GWAS Catalog format [14] (code examples available from https://github.com/mrcieu/gwas2vcf). Further, the gwasglue R package provides convenient programming functions to automate the preparation of genetic association data for a range of downstream analyses (Additional file 1: Table S2). Currently, methods exist for streamlining variant fine-mapping [37–41], colocalization [42], MR [43] and data visualisation [44]; example analytical workflows are available from https://mrcieu.github.io/gwasglue/articles. New methods are being actively added, and users may request new features via the repository issues page.

### Data resource

To encourage adoption, we made openly available over 10,000 complete GWAS summary statistics in GWAS-VCF format as part of the IEU OpenGWAS database described in a companion paper [26]. These studies include a broad range of traits, diseases and molecular phenotypes building on the initial collection for the MR Base platform [43].

## Discussion

The GWAS-VCF format has a number of advantages over existing solutions. First, the VCF provides consistent and robust approaches to storing genetic variants, annotations and metadata enabling interoperability and reusability consistent with the FAIR principles [45]. Furthermore, variable type and number requirements reduce parsing errors and missing data preventing unexpected programme operation. Second, the VCF is well established and scalable to support GWAS of whole-genome sequencing studies. Many mature tools have been developed providing a range of functions for querying, annotating, transforming and analysing genetic data in VCF. Third, the GWAS-VCF file header stores comprehensive metadata about the GWAS including necessary information to understand the analysis and interpret the data. Fourth, a GWAS-VCF file can store individual or multiple traits (in one or more sample columns) in a single file which is beneficial for the distribution of GWAS datasets where genotypes of each sample/individual have been tested for association with multiple traits (e.g. QTL datasets).

Our simulation studies demonstrated the GWAS-VCF was substantially quicker when the GWAS was densely imputed (8–44×) than TSV using standard UNIX tools for extracting records by genomic position. Although the GWAS-VCF was slower for extracting records by association *P* value, this could be improved by using variant flags (i.e. in the INFO field) to highlight records below prespecified thresholds if the exact value is unimportant. For example, all variants below genome-wide significance (*P* < 5e−8) or a more relaxed threshold (e.g. *P* < 5e−5).

A limitation of the current summary statistics formats, including GWAS-VCF, is the lack of a widely adopted and stable representation of sequence variants that can be used as a universal unique identifier for the said variants. Published summary statistics often use rsids [31] to identify variants, but this practice is inappropriate because rsids are locus identifiers and do not distinguish between multiple alternative alleles observed at the same site. Moreover, rsids are not stable as they can be merged and retired over time. The reason this is a problem is that in GWAS summary statistics, every record represents the effect of a specific allele on one or more traits, and if a record identifier is used that is not unique for each allelic substitution, it cannot technically be considered an identifier. An alternative approach is to concatenate chromosome, base position, reference and alternative allele field values into a single string, but this is non-standardised, genome build-specific and unwieldy for long insertion-deletion variants. Worst still is the common practice of mixing these types of identifiers within a single file. In version 1.2 of the GWAS-VCF specification, we suggest querying variants by chromosome and base position and filtering the output to retain the target substitution (implemented in our parsers), but we acknowledge that this approach can be cumbersome and difficult to interoperate with other software. The ideal solution would be to populate the ID column of a GWAS-VCF file using universally accepted and unique variant identifiers. We have reviewed several existing variant identifier formats as candidates for the variant identifier field, and if a consensus arises in the scientific community, it will be implemented in a future version of the specification (Additional file 1: Table S3). However, we refrain from making a unilateral choice at this juncture because successful implementation will require consultation from a range of stakeholders. The genetics community uses different approaches already to deal with the problem of sequence variant representation, and there is an urgent need to coalesce upon a single format.

Another potential limitation is the use of multiple ontologies to describe the GWAS trait which might make inter-study comparisons difficult. However, we feel enforcing a specific trait identifier system could prevent the new ontologies and non-human data which would provide a barrier to adoption.

## Conclusion

Here, we present an adaptation of the VCF specification for GWAS summary statistics storage that is amenable to high-throughput analyses and robust data sharing and integration. We implement open-source tools to convert existing summary statistics formats to GWAS-VCF, and libraries for reading or querying this format and integrating with existing analysis tools. Finally, we provide complete GWAS summary statistics for over 10,000 traits in GWAS-VCF. These resources enable convenient and efficient secondary analyses of GWAS summary statistics and support future tool development.

## Methods

### Specification

The specification was developed through the experience of collecting and harmonising GWAS summary data across two research centres at scale [43] and performing a range of representative high-throughput analyses on these data (for example, LD score regression [46], MR [47], genetic colocalisation analysis [48] and polygenic risk scores [49]).

### Query performance simulation

Densely imputed summary statistics (13,791,467 variants) for GWAS of body mass index using data from the UK Biobank were obtained from Neale et al. [35]. The data were mapped to VCF using Gwas2VCF v1.1.1 and processed using bcftools v1.10 [23] to remove multiallelic variants or records with missing dbSNP [31] identifiers. GWAS-VCF files were produced containing one or five traits by combining randomly subsampled summary statistics with either 0.5, 2.5 or 10 million variants. A tabular (unindexed) file was prepared from the GWAS-VCF to replicate a typical storage medium currently used for distributing summary statistics. Query runtime performance was compared between tabix v1.10.2 [30] and standard UNIX commands under the following conditions: single variant selection using dbSNP identifier [31] or chromosome position, multi-variant selection by association *P* value (thresholds: *P* < 5e−8, 0.2, 0.4, 0.6, 0.8) or 1-Mb genomic interval. Tests were undertaken with 100 repetitions using BGZIP [23] GWAS-VCF or unindexed TSV with and without GZIP compression on an Ubuntu v18.04 server with Intel Xeon(R) 2.0 GHz processor. All comparisons were performed using singled thread operations, and therefore, differences in runtime performance were due to tool and/or file index usage.

## Supplementary Information

**Additional file 1: Figure S1.** VCF format adapted to store GWAS summary statistics (GWAS-VCF). Example GWAS-VCF with individual sections labelled. **Table S1.** Data fields in the GWAS-VCF. Required and optional GWAS-VCF fields with descriptions as defined in the file specification. **Table S2.** Open-source tools for working with GWAS-VCF. Description of open-source software for working with GWAS-VCF and download links. **Table S3.** Possible variant identifier schemes for the ID column of GWAS-VCF. Example unique variant identifier schemes and their advantages/disadvantages.

**Additional file 2.** Review history.

## Data Availability

Open-source (MIT License) query performance evaluation source code available from Zenodo [50] or GitHub [51]. The body mass index (21001, v3) GWAS summary statistics used in the evaluation is available from http://www.nealelab.is/uk-biobank [35]. Version 1.2 of the GWAS-VCF format specification (MIT License) is available from Zenodo [52] or GitHub [53]. Full summary statistics for over 10,000 GWAS in VCF format are available from the IEU OpenGWAS Database [26] (https://gwas.mrcieu.ac.uk).
