# Supplementary material for: The variant call format provides efficient and robust storage of GWAS summary statistics
Source: Genome Biol. 2021 Jan 13;22:32. doi: 10.1186/s13059-020-02248-0 (PMC7805039; doi:10.1186/s13059-020-02248-0)
Supplement: Supplementary file 1 — Additional file 1: Figure S1. VCF format adapted to store GWAS summary statistics (GWAS-VCF). Example GWAS-VCF with individual sections labelled. Table S1. Data fields in the GWAS-VCF. Required and optional GWAS-VCF fields with descriptions as defined in the file specification. Table S2. Open-source tools for working with GWAS-VCF. Description of open-source software for working with GWAS-VCF and download links. Table S3. Possible variant identifier schemes for the ID column of GWAS-VCF. Example unique variant identifier schemes and their advantages/disadvantages. [file 13059_2020_2248_MOESM1_ESM.docx]

**Additional File 1**

Fig S1. VCF format adapted to store GWAS summary statistics (GWAS-VCF)

The GWAS-VCF file contains study and trait(s) metadata, variant-level data, and variant-trait association summary statistics. Each field is defined in the file header including variable type and number of values. The format can store the results of a GWAS with one or more traits in a single file.

Table S1. Data fields in the GWAS-VCF

| Field | Description |
| --- | --- |
| VCF Header | |
| Study | |
| ID* | Study identifier e.g. publication or data repository identifier e.g. 12345678 (PubMed) or phs001997.v1.p1 (dbGAP) |
| Source* | Source of study identifier e.g. PubMed or dbGAP |
| Version | Version of study ID source used to describe study |
| Description | Study description |
| URL | Web link to study |
| Trait | |
| ID* | Trait identifier e.g. an ontology or metadata repository identifier e.g. EFO0004340 (EFO), ieu-a-835 (IEU OpenGWAS database) or any other ontology |
| Source* | Source of trait identifier e.g. EFO [1] or IEU OpenGWAS database [2] |
| Description* | Trait description e.g. Body mass index |
| Version | Version of trait ID source used to describe trait |
| Type | Outcome variable type (continuous or binary) |
| Test | Statistical test for association data e.g. linear regression |
| Unit | Phenotype units e.g. kg/m^2^ or SD |
| Population | Participant ancestry (or mixed ancestry) using the standardised framework [3] |
| FileUrl | URL of GWAS summary statistics file |
| FileDate | Date GWAS summary statistics were produced |
| TotalSamples | Total number of samples/individuals in the study |
| TotalCases | Total number of cases in the study (if case-control) |
| TotalVariants | Total number of variants tested in the study |
| VariantsNotRead | Number of variants that could not be read |
| VariantsHarmonised | Number of harmonised variants |
| VariantsNotHarmonised | Number of variants that could not be harmonised |
| SwitchedAlleles | Number of variants strand switched |
| VCF Body | |
| Per trait variant-level information | |
| NS | Variant-specific number of samples/individuals with called genotypes used to test association with specified trait |
| EZ | Z-score provided if it was used to derive the ES and SE fields |
| SI | Accuracy score of association statistics imputation |
| NC | Variant-specific number of cases used to estimate genetic effect (binary traits only) |
| ES* | Effect size estimate relative to the alternative allele |
| SE* | Standard error of effect size estimate |
| LP* | -log10 p-value for effect estimate |
| AF | Alternative allele frequency in trait subset |
| AC | Alternative allele count in the trait subset |

ID, identifier. EFO, Experimental Factor Ontology. VCF, variant call format. * Required fields.

Table S2. Open-source tools for working with GWAS-VCF

| Program | Purpose | Implementation | Source code link |
| --- | --- | --- | --- |
| gwas2vcf* | Mapping tabular GWAS summary statistics and NHGRI-EBI GWAS Catalog format [4] to VCF | Python3 (Docker) | <https://github.com/mrcieu/gwas2vcf> |
| gwas2vcfweb* [http://vcf.mrcieu.ac.uk](http://vcf.mrcieu.ac.uk/) | Front-end and queue schedular for gwas2vcf | Python3, Cromwell [5]  (Docker) | <https://github.com/mrcieu/gwas2vcfweb> |
| gwasvcf* | Library for querying and reading GWAS-VCF files | R | <https://github.com/mrcieu/gwasvcf> |
| pygwasvcf* | Library for querying and reading GWAS-VCF files | Python3 | <https://github.com/mrcieu/pygwasvcf> |
| gwasglue* | Library for processing GWAS summary statistics ready for secondary analysis | R | <https://github.com/mrcieu/gwasglue> |
| LD Score Regression [6] (patch)† | Estimating genetic correlation and heritability | Python | <http://github.com/explodecomputer/ldsc> |

GWAS, genome-wide association study. LD, linkage disequilibrium. VCF, variant call format. NHGRI-EBI, National Human Genome Research Institute and European Bioinformatics Institute. *MIT License. †GNU General Public License v3.0. All software is designed for use on a UNIX system.

Table S3. Possible variant identifier schemes for the ID column of GWAS-VCF

| VCF row identifier (ID column) | Advantages | Disadvantages |
| --- | --- | --- |
| dbSNP [7] rsID with multiallelic variants on a single row  Example:  rs376272854 | - No duplication of information already in the row - Rsidx [8] provides fast dbSNP [7] ID queries - Widely used - Short length - Compatibility with existing tools (rsid is encouraged by VCF [9] v4.2 specification) | - Refers to a position rather than a substitution - Complexity and ambiguity of manipulating multiallelic rows - Does not distinguish between multiple alternative alleles and therefore a positional identifier - Multiple rsids can point to the same position (e.g. new dbSNP [7] entries awaiting merge with existing records) |
| No value in ID column with multiallelic variants on separate rows | - No duplication of information already in the row - Avoids the complexities of a variant identifier | - Variant queries include multiple fields (chromosome, position, reference and alternative allele) - No guarantees of row uniqueness - Difficult to operate with other software that requires a unique substitution identifier |
| HGVS [10] DNA nomenclature with multiallelic variants on separate rows  Example:  chr2:g.84918761_84918811del | - Unique identifier for every substitution - Supports one substitution per row in the VCF which is easier to parse - Short insertion-deletion encoding - Known format | - Duplicates information already stored in the row - Not stable between genome builds - Comparing between builds is difficult - Not widely used for GWAS |
| Concatenation of chromosome, position and alleles with multiallelic variants on separate rows  Example:  chr2:84918760:  CCCAACCCTGCTGTCAT  AATGCATAAGCAGCCAC  AGACAGTAAGTGAATGAA:C | - Unique identifier for every substitution - Supports one substitution per row in the VCF which is easier to parse - Known format | - Duplicates information already stored in the row - Comparing between builds is difficult - Not stable between genome builds - Long insertion-deletion coding |
| SPDI [11] (Sequence-id, Position, Deleted Sequence, Insertion Sequence separated by a colon) with multiallelic variants on separate rows  Example:  NC_000002.12: 84918760: CCCAACCCTGCTGTCAT  AATGCATAAGCAGCCAC  AGACAGTAAGTGAATGAA:C | - Unique identifier for every substitution - Supports one substitution per row in the VCF which is easier to parse   Known format | - Duplicates information already stored in the row - Comparing between builds is difficult - Not stable between genome builds - Long insertion-deletion coding |
| Concatenation of chromosome, position and alleles using MD5 hash to shorten long alleles with multiallelic variants on separate rows  Example:  chr2:84918760-7c43e7284b58ba06e  7438bff62376edf:C | - Unique (almost) identifier for every substitution - Supports one substitution per row in the VCF which is easier to parse - Short insertion-deletion coding | - Duplicates information already stored in the row - Not stable between genome builds - Comparing between builds is difficult - Cannot reverse hash without database - Not widely used |
| GA4GH Variation Representation [12] (SHA-512 message digest of the chromosome position and alternative allele with multiallelic variants on separate rows  Example:  ga4gh:VA.yOoxi7-uUnJyn4QkQ23h6RJuT4Zqarow | - Unique (almost) identifier for every substitution - Supports one substitution per row in the VCF which is easier to parse   Short insertion-deletion coding | - Duplicates information already stored in the row - Not stable between genome builds - Comparing between builds is difficult - Cannot reverse hash without database - Not widely used |

GWAS, genome-wide association study. VCF, variant call format. Rsidx, file index using the dbSNP identifier. MD5, message-digest algorithm. HGVS, Human Genome Variation Society. GA4GH, Global Alliance for Genomics and Health. SHA, Secure Hash Algorithm

**References**

1. Malone J, Holloway E, Adamusiak T, Kapushesky M, Zheng J, Kolesnikov N, et al. Databases and ontologies Modeling sample variables with an Experimental Factor Ontology. 2010 [cited 2020 Apr 21];26:1112–8. Available from: http://www.ebi.ac.uk/efo/metadata

2. Elsworth​ B, Lyon​ M, Alexander​ T, Liu​ Y, Matthews​ P, Hallett​ J, et al. The MRC IEU OpenGWAS data infrastructure. bioRxiv [Internet]. Cold Spring Harbor Laboratory; 2020 [cited 2020 Aug 13];2020.08.10.244293. Available from: https://doi.org/10.1101/2020.08.10.244293

3. Morales J, Welter D, Bowler EH, Cerezo M, Harris LW, McMahon AC, et al. A standardized framework for representation of ancestry data in genomics studies, with application to the NHGRI-EBI GWAS Catalog. Genome Biol [Internet]. BioMed Central Ltd.; 2018 [cited 2020 Apr 1];19:21. Available from: https://genomebiology.biomedcentral.com/articles/10.1186/s13059-018-1396-2

4. Buniello A, MacArthur JAL, Cerezo M, Harris LW, Hayhurst J, Malangone C, et al. The NHGRI-EBI GWAS Catalog of published genome-wide association studies, targeted arrays and summary statistics 2019. Nucleic Acids Res [Internet]. Oxford University Press; 2019 [cited 2020 Feb 18];47:D1005–12. Available from: https://academic.oup.com/nar/article/47/D1/D1005/5184712

5. Voss K, Gentry J, Auwera G Van Der. GATK4 + WDL + Cromwell. F1000Research [Internet]. 2017 [cited 2020 Feb 25];6:4. Available from: https://doi.org/10.7490/f1000research.1114631.1

6. Bulik-Sullivan B, Loh PR, Finucane HK, Ripke S, Yang J, Patterson N, et al. LD score regression distinguishes confounding from polygenicity in genome-wide association studies. Nat Genet. 2015;

7. Sherry ST, Ward M-H, Kholodov M, Baker J, Phan L, Smigielski EM, et al. dbSNP: the NCBI database of genetic variation [Internet]. Nucleic Acids Res. 2001. Available from: http://www.ncbi.nlm.nih.gov/SNP.

8. Standage D. Library for indexing VCF files for random access searches by rsID. GitHub. https://github.com/bioforensics/rsidx (2020)

9. Danecek P, Auton A, Abecasis G, Albers CA, Banks E, DePristo MA, et al. The variant call format and VCFtools. Bioinformatics. 2011;27:2156–8.

10. den Dunnen JT, Dalgleish R, Maglott DR, Hart RK, Greenblatt MS, McGowan-Jordan J, et al. HGVS Recommendations for the Description of Sequence Variants: 2016 Update. Hum Mutat [Internet]. John Wiley and Sons Inc.; 2016 [cited 2020 May 5];37:564–9. Available from: http://doi.wiley.com/10.1002/humu.22981

11. Holmes JB, Moyer E, Phan L, Maglott D, Kattman B. SPDI: data model for variants and applications at NCBI. [cited 2020 May 5]; Available from: https://api.ncbi.nlm.nih.gov/variation/v0

12. Babb L, Wagner AH, Schuilenburg H, Cline M, Riehle K, Lee J, et al. ga4gh/vr-spec: 1.1. 2020 [cited 2020 Dec 10]; Available from: https://zenodo.org/record/4021714
