# Supplementary material for: The variant call format provides efficient and robust storage of GWAS summary statistics
Source: Genome Biol. 2021 Jan 13;22:32. doi: 10.1186/s13059-020-02248-0 (PMC7805039; doi:10.1186/s13059-020-02248-0)
Supplement: Supplementary file 2 — Additional file 2. Review history. [file 13059_2020_2248_MOESM2_ESM.docx]

Review History

**First round of review**

**Reviewer 1**

**Were you able to assess all statistics in the manuscript, including the appropriateness of statistical tests used?**

No

**Were you able to directly test the methods?**

No

**Comments to author:**

The manuscript describes a new file format proposed as a community standard for GWAS summary statistics. Standardization is a pressing issue for the GWAS community, and this new format is a welcome addition to the existing solutions listed in the paper, such as that offered by the GWAS Catalog. In addition to the format, the authors have also developed and described tools to process, handle and support interoperability of GWAS-VCF files, and demonstrate faster query times of GWAS-VCF over tabular files. The manuscript is well written and clear, with appropriate rationale and discussion. The problems surrounding use of rs IDs for variant identification are well described.

Some suggestions for improvement are listed below.

The analysis would be improved by the use of more than one example. The densely imputed summary statistics chosen for analysis are on the larger end of typical GWAS data. How does the query performance comparison look when handling smaller files (which are the most popular/shared files)—is the query response more similar to TSV files? Does the VCF file mostly make the difference for densely imputed files? How does the comparison look when querying a file containing single vs multiple traits?

It would be useful to expand on the use of trait identifiers in the format. The practicalities of how trait information is handled in the VCF format are not clear.

Firstly, multiple ontologies are listed as examples, but it is not clear whether any ontology can be used or only the 4 listed in T1. Is there any requirement for one ontology to be used in preference to others? For the example given in TS1 (BMI can be represented as either EFO0004340 or ieu-a-835), if different datasets use different ontologies, the user is required to do some ontology mapping in order to pull together multiple GWAS on the same trait, which could be a limitation. This is worthy of further discussion in the manuscript.

Secondly, trait ID is a mandatory field, but some novel, complex or highly specific traits may not be easily (or at all) represented by a single standard ontology term. With over 10,000 datasets, the authors have most likely encountered less straightforward traits, and it would be useful to hear how they have been dealt with. Regarding the format itself, trait description should also be a required field, otherwise important experimental detail may be lost.

The authors could elaborate more on the limitations re use of GWAS-VCF to report results from whole-genome sequencing studies and describe actual plans to face the limitation in next release of format. Would adopting GA4GH standards address the issue? While TS2 contains plenty of details on advantages and disadvantages of possible nomenclature standards, the higher level solution should be discussed further in "discussion"

The gold standard for scientific data management is described by the FAIR principles (Wilkinson et al 2016), developed to overcome obstacles in data discovery and re-use. The GWAS-VCF format goes some way towards ensuring that GWAS summary statistics meet these principles, and the manuscript could be improved by a brief discussion of this.

Little mention is made of issues around the use of different reference genome assemblies. Have the authors considered coercing all data to one genome build? It would be worth noting that there are some well established tools for performing liftover to VCF files.

On line 165-166, please expand on the conversion process and how it can be performed, since this is likely to be of interest to readers.

In the section entitles "Software", first paragraph, will you need to mandate zero or one-based variant calling?

On line 208-209, a further downside of concatenating chr, bp and alleles is that large indels can present a problem.

In table 1, the claim that the file content is easily human-readable is not borne out by the example shown in fig 1, and should be toned down.

Please indicate the source of the 10,000 datasets described in the paper eg similar to the table found in https://gwas.mrcieu.ac.uk/datasets/. Are there plans to make more data available in the future?

Throughout the paper, please be consistent in terminology when referring to the GWAS Catalog's standard format as the "NHGRI-EBI GWAS Catalog format" and cite the appropriate paper, ref 14.

**Reviewer 2**

**Were you able to assess all statistics in the manuscript, including the appropriateness of statistical tests used?**

Yes: all statistics appear to be sound.

**Were you able to directly test the methods?**

No

**Comments to author:**

General comments: Lyon and coauthors developed a new format for GWAS summary statistics based on the VCF file format commonly used for individual-level sequencing data. In addition, a suite of Python libraries and R packages have been developed to serve as the interface between association analysis software such as plink and GCTA and downstream post-GWAS analysis such as LDpred and Mendelian randomization analysis. The authors have also made the efforts to convert a large number of published GWAS summary statistics to the proposed GWAS-VCF format, available on the authors' website, which is a very helpful. While the technical advancement is sound and some caveats with the current summary statistics formats are well recognized by the authors, the computational speed-up seems to be quite marginal and some problems with summary statistics remain unsolved. Moreover, the GWAS-VCF appeared to be a relatively straightforward extension of the VCF format, compromising the proposed format's novelty. I have the following specific comments.

1. Figure 2 is not referenced in the main text. On page 6, line 136, the authors stated that "Simulations of query performance demonstrate compressed GWAS-VCF is substantially quicker than unindexed and uncompressed TSV format for querying by genomic position." However, based on Figure 2, the mean runtime for querying text and the proposed GWAS-VCF was quite close to each other. For some applications (e.g., p-value-based), the GWAS-VCF-based querying appeared to be slower.

2. Does the current software implementation have functions to support reference genome version and/or gene/pathway annotation?

3. A showcase of a real data example would be appreciated.

**Authors Response**

**Point-by-point responses to the reviewers’ comments:**

Reviewer 1:

The manuscript describes a new file format proposed as a community standard for GWAS summary statistics. Standardization is a pressing issue for the GWAS community, and this new format is a welcome addition to the existing solutions listed in the paper, such as that offered by the GWAS Catalog. In addition to the format, the authors have also developed and described tools to process, handle and support interoperability of GWAS-VCF files, and demonstrate faster query times of GWAS-VCF over tabular files. The manuscript is well written and clear, with appropriate rationale and discussion. The problems surrounding use of rs IDs for variant identification are well described.

The analysis would be improved by the use of more than one example. The densely imputed summary statistics chosen for analysis are on the larger end of typical GWAS data. How does the query performance comparison look when handling smaller files (which are the most popular/shared files)—is the query response more similar to TSV files? Does the VCF file mostly make the difference for densely imputed files? How does the comparison look when querying a file containing single vs multiple traits?

*A: Thank you for your comments. We have included example comparisons with 0.5, 2.5 and 10 million variants. We also compared storing single vs multiple traits in a single GWAS-VCF file.*

*Changes: Please see Figure 1.*

It would be useful to expand on the use of trait identifiers in the format. The practicalities of how trait information is handled in the VCF format are not clear.

Firstly, multiple ontologies are listed as examples, but it is not clear whether any ontology can be used or only the 4 listed in T1. Is there any requirement for one ontology to be used in preference to others? For the example given in TS1 (BMI can be represented as either EFO0004340 or ieu-a-835), if different datasets use different ontologies, the user is required to do some ontology mapping in order to pull together multiple GWAS on the same trait, which could be a limitation. This is worthy of further discussion in the manuscript.

*A: The format is flexible and can accommodate any ontology. We do not wish to impose specific requirements so that the format can be used flexibly for example with newly developed ontologies or non-human data. We have updated the manuscript to make this clearer and also described the limitations of working with multiple ontologies in the discussion.*

*Changes:*

*- Table 1, “The file header contains information about the source and date of summary statistics, study IDs (e.g., PMID/DOI of publication describing the study, or accession number and repository of individual-level data), description of the trait(s) studied (e.g., type, association test used, and measurement unit) as well as the source and version of trait IDs (e.g., IEU OpenGWAS database [35], Experimental Factor Ontology [17], Human Phenotyping Ontology [18], Medical Subject Headings [19] IDs for clinical and other traits, Ensembl Gene IDs for eQTL datasets, or any other ontology suitable to describe the data).”*

*- Supplementary Table 2, “Trait identifier e.g. an ontology or metadata repository identifier e.g. EFO0004340 (EFO), ieu-a-835 (IEU OpenGWAS database) or any other ontology“*

*- Lines 112-115, “as well as the source and version of trait IDs (e.g., Experimental Factor Ontology [17], Human Phenotyping Ontology [18], Medical Subject Headings [19] IDs for clinical and other traits, Ensembl Gene IDs for eQTL datasets, or any other ontology).”*

*- Lines 240-243, “Another potential limitation is the use of multiple ontologies to describe the GWAS trait which might make inter-study comparisons more difficult. However, we felt enforcing a specific system could prevent the use of future ontologies and non-human data which would provide a barrier to adoption.”*

Secondly, trait ID is a mandatory field, but some novel, complex or highly specific traits may not be easily (or at all) represented by a single standard ontology term. With over 10,000 datasets, the authors have most likely encountered less straightforward traits, and it would be useful to hear how they have been dealt with. Regarding the format itself, trait description should also be a required field, otherwise important experimental detail may be lost.

*A: We are able to accommodate complex phenotypes by describing the trait using a specific ontology, for example GWAS of UK Biobank traits are described using UK Biobank field identifiers. We made trait description a required field.*

*Changes: Supplementary Table 2.*

The authors could elaborate more on the limitations re use of GWAS-VCF to report results from whole-genome sequencing studies and describe actual plans to face the limitation in next release of format. Would adopting GA4GH standards address the issue? While TS2 contains plenty of details on advantages and disadvantages of possible nomenclature standards, the higher level solution should be discussed further in "discussion"

*A: The GWAS-VCF is fully supportive of results from whole genome-sequencing studies. We have updated the text accordingly to clarify this point. The proposed GWAS-VCF format is agnostic in regard to the choice of variant ID nomenclature standard and we feel that proposing a higher-level solution is beyond the scope of this short report and research group. We however felt it was important to highlight the limitations of standard practices (e.g., the use of rsIDs) and the need for a widely accepted standard. The advantage of using GWAS-VCF is that the variant itself is fully documented (in terms of genome build, chromosome, position and ref/alt alleles) and thus applying a different ID scheme becomes a simple re-annotation task.*

*Changes: lines 196-197 “Second, the VCF is well established and scalable to support GWAS of whole-genome sequencing studies.”*

The gold standard for scientific data management is described by the FAIR principles (Wilkinson et al 2016), developed to overcome obstacles in data discovery and re-use. The GWAS-VCF format goes some way towards ensuring that GWAS summary statistics meet these principles, and the manuscript could be improved by a brief discussion of this.

*A: We have described how the format is consistent with the FAIR principles.*

*Changes: lines 193-201 “First, the VCF provides consistent and robust approaches to storing genetic variants, annotations and metadata enabling interoperability and reusability consistent with the FAIR principles [36]… Third, the GWAS-VCF file header stores comprehensive metadata about the GWAS including necessary information to understand the analysis and interpret the data”*

Little mention is made of issues around the use of different reference genome assemblies. Have the authors considered coercing all data to one genome build? It would be worth noting that there are some well established tools for performing liftover to VCF files.

*A: We have made reference to the use of liftover and provided code examples.*

*Changes: lines 171-175 “These tools also enable variant annotation and filtering (e.g. allele frequency, functional effect, gene and pathway), mapping between reference genome assemblies, file validation and converting to any other tabular format including the NHGRI-EBI GWAS Catalog format [14] (code examples available from: github.com/mrcieu/gwas2vcf).”*

On line 165-166, please expand on the conversion process and how it can be performed, since this is likely to be of interest to readers.

*A: We have provided code examples on how this can be achieved.*

*Changes: lines 169-175 “Once stored in a GWAS-VCF file, summary statistics can be read and queried using R or Python programming languages with our open-source libraries (Supplementary Table 1) or from the command line using for example: bcftools [24], GATK [25] or bedtools [26]. These tools also enable variant annotation and filtering (e.g. allele frequency, functional effect, gene and pathway), mapping between reference genome assemblies, file validation and converting to any other tabular format including the NHGRI-EBI GWAS Catalog format [14] (code examples available from: github.com/mrcieu/gwas2vcf).”*

In the section entitles "Software", first paragraph, will you need to mandate zero or one-based variant calling?

*A: We have made the requirement for 1-based variant calling explicit.*

*Changes: lines 157-159 “The application reads in metadata and variant-trait association data using a user-defined schema requiring the chromosome base-position to start at one”*

On line 208-209, a further downside of concatenating chr, bp and alleles is that large indels can present a problem.

*A: Thank you for the suggestion, we have added to the text.*

*Changes: lines 222-225 “An alternative approach is to concatenate chromosome, base-position, reference and alternative allele field values into a single string, but this is non-standardised, genome build specific and unwieldy for long insertion-deletion variants”.*

In table 1, the claim that the file content is easily human-readable is not borne out by the example shown in fig 1, and should be toned down.

*A: By “easily human-readable” we meant to say that GWAS-VCF not being a binary format but rather a text file (a tab-delimited ascii/unicode table with headers and comment lines) that can be easily opened and read by humans either by eye (using standard text editors or command line tools) or by writing scripts (using standard text parsing functions/libraries).*

*Changes: Table 1, ‘Easily read with any text editor’ -> ‘Read with any text viewer’*

Please indicate the source of the 10,000 datasets described in the paper eg similar to the table found in https://gwas.mrcieu.ac.uk/datasets/. Are there plans to make more data available in the future?

*A: We have added reference to our companion paper describing the resource in detail. Yes, we plan to make new data available.*

*Changes: lines 185-187, “To encourage adoption, we made openly available over 10,000 complete GWAS summary statistics in GWAS-VCF format as part of the IEU OpenGWAS database described in a companion paper [35].”*

Throughout the paper, please be consistent in terminology when referring to the GWAS Catalog's standard format as the "NHGRI-EBI GWAS Catalog format" and cite the appropriate paper, ref 14.

*A: We have updated the text accordingly.*

*Changes: lines 72, 174, Supplementary Table 1.*

Reviewer 2:

General comments: Lyon and coauthors developed a new format for GWAS summary statistics based on the VCF file format commonly used for individual-level sequencing data. In addition, a suite of Python libraries and R packages have been developed to serve as the interface between association analysis software such as plink and GCTA and downstream post-GWAS analysis such as LDpred and Mendelian randomization analysis. The authors have also made the efforts to convert a large number of published GWAS summary statistics to the proposed GWAS-VCF format, available on the authors' website, which is a very helpful. While the technical advancement is sound and some caveats with the current summary statistics formats are well recognized by the authors, the computational speed-up seems to be quite marginal and some problems with summary statistics remain unsolved.

*A: Thank you for your comments. In the original version of the manuscript Figure 2 reported query performance using the log10 scale because the improvements using GWAS-VCF were very large, for example we reported GWAS-VCF was 16x faster to extract a single variant using chromosome position (mean query duration in GWAS-VCF 0.08 seconds [95% CI 0.08, 0.08]) vs mean query duration in TSV 1.29 seconds [95% CI 1.29, 1.30]) and 9x quicker using the rsid (0.09 seconds [95% CI 0.09, 0.09] vs 0.81 seconds [95% 0.80, 0.82]). Using a 1Mb window of variants GWAS-VCF was 46x quicker (0.11 seconds [95% CI 0.11, 0.11] vs 5.02 seconds [95% CI 4.99, 5.04]). Additionally, given the scale of our data (i.e. > 125 billion variants from >34,000 analyses) even a modest increase in performance would offer huge benefit.*

*Changes: Figure 1 shows performance improvements in seconds using the natural scale.*

Moreover, the GWAS-VCF appeared to be a relatively straightforward extension of the VCF format, compromising the proposed format's novelty.

*A: We feel adopting an existing format is a positive solution to the problem. This approach lowers the barrier for adoption because users are already familiar with existing compatible tools and resources.*

I have the following specific comments.

1. Figure 2 is not referenced in the main text. On page 6, line 136, the authors stated that "Simulations of query performance demonstrate compressed GWAS-VCF is substantially quicker than unindexed and uncompressed TSV format for querying by genomic position." However, based on Figure 2, the mean runtime for querying text and the proposed GWAS-VCF was quite close to each other. For some applications (e.g., p-value-based), the GWAS-VCF-based querying appeared to be slower.

*A: Reference to the figure has been added. Please see above response, the differences in query time between TSV and GWAS-VCF for genomic location were reported using a log10 scale because the difference was very large. We have switched to natural scale to improve interpretability.*

*Changes: Please see Figure 1.*

2. Does the current software implementation have functions to support reference genome version and/or gene/pathway annotation?

*A: Yes, we have added code examples.*

*Changes: lines 171-175 “These tools also enable variant annotation and filtering (e.g. allele frequency, functional effect, gene and pathway), mapping between reference genome assemblies, file validation and converting to any other tabular format including the NHGRI-EBI GWAS Catalog format [14] (code examples available from: github.com/mrcieu/gwas2vcf).”*

3. A showcase of a real data example would be appreciated.

*A: We have produced example analytical workflows to demonstrate the utility of GWAS-VCF.*

*Changes: lines 177-180 “Currently, methods exist for streamlining variant fine-mapping [27–31], colocalization [32], MR [33] and data visualisation [34], example analytical workflows are available from: https://mrcieu.github.io/gwasglue/articles”.*

**Second round of review**

**Reviewer 1**

The manuscript has been improved in particular by the addition of more detailed analysis and examples of query times, and all points have been adequately addressed.
